# Andexanet alfa effectively reverses edoxaban anticoagulation effects and associated bleeding in a rabbit acute hemorrhage model

**DOI:** 10.1371/journal.pone.0195122

**Published:** 2018-03-28

**Authors:** Genmin Lu, Polly Pine, Janet M. Leeds, Francis DeGuzman, Pratikhya Pratikhya, Joyce Lin, John Malinowski, Stanley J. Hollenbach, John T. Curnutte, Pamela B. Conley

**Affiliations:** Portola Pharmaceuticals Inc., South San Francisco, CA, United States of America; University of Pennsylvania Perelman School of Medicine, UNITED STATES

## Abstract

**Introduction:**

Increasing use of factor Xa (FXa) inhibitors necessitates effective reversal agents to manage bleeding. Andexanet alfa, a novel modified recombinant human FXa, rapidly reverses the anticoagulation effects of direct and indirect FXa inhibitors.

**Objective:**

To evaluate the ability of andexanet to reverse anticoagulation *in vitro* and reduce bleeding in rabbits administered edoxaban.

**Materials and methods:**

*In vitro* studies characterized the interaction of andexanet with edoxaban and its ability to reverse edoxaban-mediated anti-FXa activity. In a rabbit model of surgically induced, acute hemorrhage, animals received edoxaban vehicle+andexanet vehicle (control), edoxaban (1 mg/kg)+andexanet vehicle, edoxaban+andexanet (75 mg, 5-minute infusion, 20 minutes after edoxaban), or edoxaban vehicle+andexanet prior to injury.

**Results:**

Andexanet bound edoxaban with high affinity similar to FXa. Andexanet rapidly and dose-dependently reversed the effects of edoxaban on FXa activity and coagulation pharmacodynamic parameters *in vitro*. In edoxaban-anticoagulated rabbits, andexanet reduced anti-FXa activity by 82% (from 548±87 to 100±41 ng/ml; *P*<0.0001), mean unbound edoxaban plasma concentration by ~80% (from 100±10 to 21±6 ng/ml; *P*<0.0001), and blood loss by 80% vs. vehicle (adjusted for control, 2.6 vs. 12.9 g; *P* = 0.003). The reduction in blood loss correlated with the decrease in anti-FXa activity (r = 0.6993, *P*<0.0001) and unbound edoxaban (r = 0.5951, *P* = 0.0035).

**Conclusion:**

These data demonstrate that andexanet rapidly reversed the anticoagulant effects of edoxaban, suggesting it could be clinically valuable for the management of acute and surgery-related bleeding. Correlation of blood loss with anti-FXa activity supports the use of anti-FXa activity as a biomarker for assessing anticoagulation reversal in clinical trials.

## Introduction

Direct factor Xa (FXa) inhibitors are increasingly being used as anticoagulant therapy for the management of thromboembolic disorders such as prevention of stroke and systemic embolism in patients with non-valvular atrial fibrillation, prophylaxis/treatment of venous thromboembolism, and thromboprophylaxis following hip or knee replacement surgery. This class of drugs offers many advantages over vitamin K antagonists in patients with non-valvular atrial fibrillation, including a lower risk of stroke, systemic embolic events, and mortality, as well as a reduction in major bleeding events [1, 2]. Direct FXa inhibitors also have a more rapid onset of action, fewer drug-drug interactions, and more predictable pharmacokinetics compared with vitamin K antagonists, thus eliminating the necessity for frequent monitoring of coagulation parameters [3]. Since their introduction in 2011, global use of these agents continues to increase [3, 4]. However, like all anticoagulants, FXa inhibitors are associated with a risk of bleeding, with annual major bleeding rates ranging from 1.6% to 3.6% of the patient population [5–7]. While idarucizumab (Praxbind^®^) was recently approved as a reversal agent for dabigatran, a direct thrombin inhibitor, there is no approved reversal agent for FXa inhibitors.

Clotting factor replacement with 3- and 4-factor prothrombin complex concentrates (PCCs) has been investigated as a means of reversing FXa inhibition [8–21]. However, PCCs are not specific reversal agents for FXa inhibitors as these agents were developed either to replace clotting factors in hemophilia or for patients being treated with vitamin K antagonists where levels of factors VII, IX, X, and II are greatly reduced.

Andexanet alfa (andexanet) is a novel, specific reversal agent that has demonstrated in preclinical studies to rapidly reverse the anticoagulation effects of direct and indirect FXa inhibitors, including rivaroxaban, apixaban, betrixaban, fondaparinux, and enoxaparin [22]. Andexanet is a modified, recombinant, human FXa that is enzymatically inactive, while retaining the ability to bind to direct and indirect FXa inhibitors [22]. Importantly, andexanet lacks the membrane-binding γ-carboxyglutamic acid domain of native FXa, and therefore does not compete with native FXa for assembly into the prothrombinase complex. Early *in vitro* studies using purified enzyme systems showed that andexanet dose-dependently reversed the anti-FXa activity of betrixaban, rivaroxaban, and apixaban [22]. In an ongoing study, andexanet is being evaluated for reversal of FXa anticoagulation, hemostatic efficacy, and clinical safety in patients with acute major bleeding [23, 24].

Edoxaban has been approved for reduction in the risk of stroke and for treatment of deep vein thrombosis (DVT) and pulmonary embolism (PE). It is crucial to better characterize and understand andexanet’s ability to reverse the anticoagulation effects of edoxaban compared with other FXa inhibitors (rivaroxaban, apixaban, betrixaban, and enoxaparin), which will inform correct clinical dosing regimens for andexanet to reverse the anticoagulation effects of edoxaban in bleeding patients. Therefore, additional *in vitro* studies were conducted to characterize the interaction of andexanet with edoxaban, and to investigate its ability to reverse edoxaban-mediated anti-FXa activity. Subsequently, a rabbit liver laceration model of acute hemorrhage was used to evaluate the ability of andexanet to reduce blood loss and normalize coagulation pharmacodynamic (PD) parameters following the administration of edoxaban.

## Materials and methods

### *In vitro* characterization of edoxaban-andexanet interaction

#### FXa enzymatic activity assay

FXa (Hematologic Technologies) was mixed with edoxaban (Daiichi Sankyo) in the absence or presence of varying andexanet concentrations in buffer conditions (20 mM Tris, 150 mM NaCl, 5 mM Ca^2+^, 0.1% BSA, pH = 7.4). In a total 200-μL reaction mixture volume, inhibition of FXa by edoxaban (Ki) in the absence of andexanet was measured with FXa at 0.5 and 1.0 nM, and with increasing concentrations of edoxaban (0–12 nM). Binding of edoxaban to andexanet (Kd) was measured with 3.0 nM FXa, 0, 2.5, 5.0, and 7.5 nM edoxaban, and increasing concentrations of andexanet (0–500 nM). Following a 2-hour incubation at room temperature, residual FXa activity was measured by cleavage of the FXa peptidyl substrate (100 μM), Spectrozyme-FXa (American Diagnostica) in a kinetic plate reader (Molecular Devices, Sunnyvale, CA). Initial rates of peptidyl substrate hydrolysis were determined by continuously monitoring A_405_ at room temperature over 5 minutes. For fitting of kinetic data, initial rates (mOD_405_/min) were converted to molar concentration terms using E_405_ = 9887 M^-1^·cm^-1^, and an effective path length of 0.59 cm for a 200-μL reaction volume [25].

For the inhibition of FXa by edoxaban, the initial rates with 0.5 nM and 1.0 nM FXa were fitted globally with Dynafit (version 4, BioKin) according to the equations describing reversible competitive inhibition of FXa by edoxaban. Ki (nM) and kcat (1/s) were obtained from the fitting using a fixed Km (Km = 82.2 μM) for Spectrazyme-Xa pre-determined in a separate experiment.

Similarly, the initial rates in the Kd measurements with 0, 2.5, 5.0, and 7.5 nM edoxaban were fitted globally with Dynafit, with inclusion of an additional equilibrium equation describing reversible binding of edoxaban to andexanet. Kd (nM) and kcat were obtained from the fitting using fixed, pre-determined Km and Ki as described above in order to reduce the number of parameters to be estimated in Dynafit.

Each experiment was repeated three times under the same conditions. The kinetic parameters (Ki, Kd) from each experiment (a, b, c) were reported along with the mean values (±standard deviations).

#### Reversal of edoxaban anti-FXa activity

Edoxaban (74 ng/mL, 136 nM) was added to pooled human plasma (BioReclamation), and andexanet was prepared in Tris-buffered saline as a 3X concentrated stock solution. Andexanet stock (25 μL) was mixed with edoxaban-containing plasma (75 μL) in a 96-well plate and incubated at room temperature for 30 minutes, followed by assessment of residual FXa activity using a modified anti-FXa assay. The reagents from a commercial heparin kit (Coamatic, DiaPharma Group, Inc) were reconstituted as a 2X stock solution for FXa substrate S2732 and 1X stock solution for bovine FXa, respectively. FXa substrate (25 μL) was first added to the wells and incubated for 15 minutes. The reaction was initiated by adding bovine FXa (25 μL) and stopped after 5 minutes by adding 20% acetic acid (50 μL). The absorbance (405 nm) was read in a plate reader (Molecular Devices, Sunnyvale, CA) and the results were calculated by a 4-parameter equation after background subtraction. Known edoxaban concentrations of 0 to 100 ng/mL prepared in plasma were used to construct a standard curve for each 96-well plate. The edoxaban anti-FXa activity was expressed as ng/mL.

#### Prothrombin time and activated partial thromboplastin time

Edoxaban and andexanet were prepared separately in pooled human plasma (BioReclamation) as 2X concentrated stocks. Equal volumes of edoxaban and andexanet stock were mixed to yield the final assay concentrations. Both assays were measured on a Stago STA Compact instrument according to the manufacturer's instructions, with 50 μL of human plasma in a total reaction volume of 120 μL.

#### Tissue factor (TF)-initiated thrombin generation assay

Edoxaban and andexanet were prepared separately in pooled human plasma (Precision Biologic) as 2X concentrated stocks. Equal volumes of edoxaban and andexanet stock were mixed to yield the final assay concentrations. Tris-buffered saline was used as a control for andexanet. The assays were performed on a calibrated automated thrombogram instrument according to the manufacturer’s instructions using the PPP-reagent (5 pM TF) (Diagnostica Stago), with 80 μL of plasma in a final reaction volume of 120 μL per well in triplicate.

### Rabbit acute hemorrhage model

Study procedures were completed in compliance with the “Guide for the Care and Use of Laboratory Animals” (National Research Council, eighth edition, 2011) and with prior approval from Portola’s Institutional Animal Care and Use Committee.

#### Study agents

Andexanet (Portola Pharmaceuticals) was stored frozen at –80°C. The frozen material was thawed in the refrigerator for 24 hours and allowed to warm to room temperature for 2 to 3 hours prior to use. Andexanet (3 mg/mL) or matching vehicle control was administered intravenously at a rate of 5 mL/minute over 5 minutes into the marginal ear vein (total, 75 mg/rabbit). The andexanet vehicle control solution was composed of Tris (10 mM, pH 7.8), L-arginine HCl (95 mM), sucrose (4%), and Tween 80 (0.01%).

Edoxaban (Daiichi Sankyo) was reconstituted in vehicle solution and administered to rabbits as a 1 mg/kg intravenous (IV) bolus injection over approximately 30 to 60 seconds into the marginal ear vein. The edoxaban dose was selected based on data from previous rabbit pharmacokinetic studies that determined the dose required to cause approximately a 2-fold increase in blood loss compared with vehicle. The edoxaban vehicle solution was composed of 90%/8%/2% v/v PEG 300/H_2_0/DMSO.

#### Animals

Male New Zealand White rabbits (Charles River, St. Constance, QC, Canada) approximately 3-months old (2.7 ± 0.07 kg) were used. Animals were acclimated for at least 72 hours in house prior to study in individual wire-steel cages under a 12-hour/12-hour light−darkness cycle. Animals were fed *ad libitum* and had free access to tap water.

#### Study design

**Fig 1:**
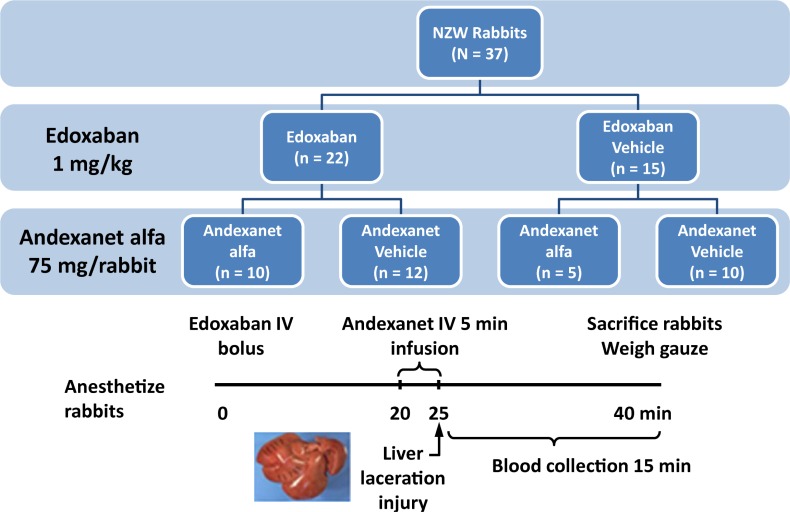
Design of rabbit liver laceration model of acute hemorrhage study.

This prophylaxis model, where liver injury occurs after administration of andexanet, was designed to mimic a “real world” situation where reversal of anticoagulation would be required prior to a surgical intervention. As shown in Fig 1, rabbits were divided into 2 anticoagulation dosing groups: edoxaban or edoxaban vehicle. Edoxaban (1 mg/mL) or vehicle was administered at Time 0. Each group was further divided into 2 reversal groups: andexanet or andexanet vehicle. Andexanet (3 mg/mL) or andexanet vehicle was administered via 5-minute infusion from Time 20 to 25 minutes. The catheter was flushed with saline to ensure complete delivery of study agents.

#### Study endpoints

The ability of andexanet to reverse the anticoagulant effects of edoxaban was evaluated by volume of blood loss and PD markers. PD markers included anti-FXa activity, PT, and aPTT. The total and pharmacologically active unbound concentration of edoxaban, as well as andexanet plasma concentrations, were also determined. Only the free fraction (not bound to plasma proteins or andexanet) of the FXa inhibitor is pharmacologically active. As such, a decrease in unbound plasma concentration corresponds to a decrease in the pharmacologically active inhibitor.

#### Procedure

The procedure for the rabbit liver injury prophylactic blood loss model was a modification [22] of a previously published hepatosplenic liver injury model [16, 17], but no injury to the spleen was performed. Briefly, rabbits were anesthetized by intramuscular administration of ketamine (44 mg/kg), xylazine (6.25 mg/kg), and butorphanol (0.125 mg/kg). Intravenous anesthesia with intramuscular maintenance administration was used. Rabbits were positioned on a circulating water-heating blanket to maintain core body temperature within a range of 36.1°C to 39.4°C; monitored by rectal thermometer. Just prior to laparotomy, an additional 0.2 mL or more of the anesthetic cocktail was administered intravenously to achieve a surgical plane of anesthesia. Marginal ear veins were catheterized with a 22G IntraCath™ catheter (BD Biosciences, Sandy, UT) for IV administration of study agents. A 20G IntraCath™ catheter was implanted surgically in the left femoral vein for serial blood sampling.

A laparotomy was performed 15 minutes after edoxaban (or edoxaban vehicle) bolus administration. Lower lobes of the liver were isolated with pre-weighed dry gauze. At Time 20 minutes (20 minutes after administration of edoxaban or edoxaban vehicle), andexanet or andexanet vehicle was administered IV over 5 minutes. A standardized injury (10 1-cm–long and 2- to 3-mm–deep incisions) was made into 2 liver lobes with 5 incisions in each lobe and allowed to bleed for 15 minutes (from Time 25 to Time 40 minutes). The gauze was removed, and any additional blood loss in the peritoneal cavity was collected with additional pre-weighed dry gauze. The two sets of gauze were combined and weighed immediately. The technician conducting the surgery was blinded to the treatment group.

#### Sample collection

Blood samples were collected at Time 0 (baseline, prior to edoxaban/edoxaban vehicle administration), 20 minutes (prior to andexanet/andexanet vehicle administration), 25 minutes (after andexanet/andexanet vehicle administration), and 40 minutes (after 15 minutes of bleeding). Each sample was 2.0 mL anticoagulated with 3.2% sodium citrate (1 part citrate to 9 parts whole blood). Platelet-poor plasma was prepared by centrifugation of the blood (5 minutes at 1814 ×*g*) and aliquoted for analysis of study agent concentrations and PD measurements.

### Rabbit model assessments

#### Blood loss

Volume of blood loss was expressed as the net weight of blood (in grams) on all the gauze material collected at study end.

#### Edoxaban plasma levels (total and unbound)

Edoxaban plasma levels were determined by liquid chromatography tandem mass spectrometry (LC/MS/MS) with a turbo-ion spray source (Shimadzu LC-20AD with Sciex API5500) using an internal standard (edoxaban-D6). Pharmacologically active unbound edoxaban was separated using an HTD96b apparatus (HTDialysis, LLC, Gales Ferry, CT), at 37°C for approximately 4 hours with gentle vortexing. Please see **S1 File** for additional details.

#### Andexanet plasma levels

Andexanet plasma levels were determined by an enzyme-linked immunosorbent assay (ELISA) with paired antibodies recognizing human FX/FXa (Enzyme Research Laboratories, Cat# FX-EIA). Andexanet standard was freshly prepared in blocking buffer with the same lot material used in this study for 8-point curve (0–200 ng/mL). The absorbance at 450 nm (OD_450_) was read by a 96-well plate reader (Molecular Devices), and the results were calculated by a 4-parameter equation (Softmax Pro 5.4, Molecular Devices).

#### Pharmacodynamic parameters

Anti-FXa activity was measured by a modified assay using reagents from a commercial Heparin kit (Coamatic, DiaPharma), as described above for human plasma, substituting pooled rabbit plasma and using edoxaban as the standard. The anti-FXa activity in the plasma samples was calculated from the edoxaban standard curve and is expressed as ng/mL. Determination of PT and aPTT was performed on an Instrumentation Laboratory ACL Elite; PT using a HemosIL PT-Fibrinogen kit and aPTT using a HemosIL SynthASil kit (both kits from Instrumentation Laboratory).

#### Correlations

Total blood loss was determined for each individual rabbit and plotted against the plasma anti-FXa activity or pharmacologically active unbound edoxaban plasma concentration from the same rabbit. Plasma samples for correlation of anti-FXa activity and unbound edoxaban concentration vs. blood loss were collected immediately following the end of andexanet administration (Time 25 minutes).

### Statistical analysis

*In vitro* FXa enzymatic activity assay and the data derived from the individual reversal kinetics were reported along with the mean values (± standard deviations).

Data from the rabbit model were plotted as mean ± standard deviation. Data were analyzed by Student unpaired *t*-test comparing each treatment group to its appropriate vehicle/medium group (two-tail distribution, homoscedastic) and confirmed using nonparametric analysis (Wilcoxon rank test) for blood loss, PT, aPTT, and anti-FXa activity. Non-parametric analysis of data from individual rabbits, Spearman correlation coefficient (r), was used for assessing correlations between blood loss and plasma anti-FXa activity or pharmacologically active unbound edoxaban concentration (SAS 9.3, Cary, NC) and confirmed using Kendall’s tau. Statistical significance was defined as *P* ≤ 0.05.

## Results

### *In vitro* studies

**Fig 2:**
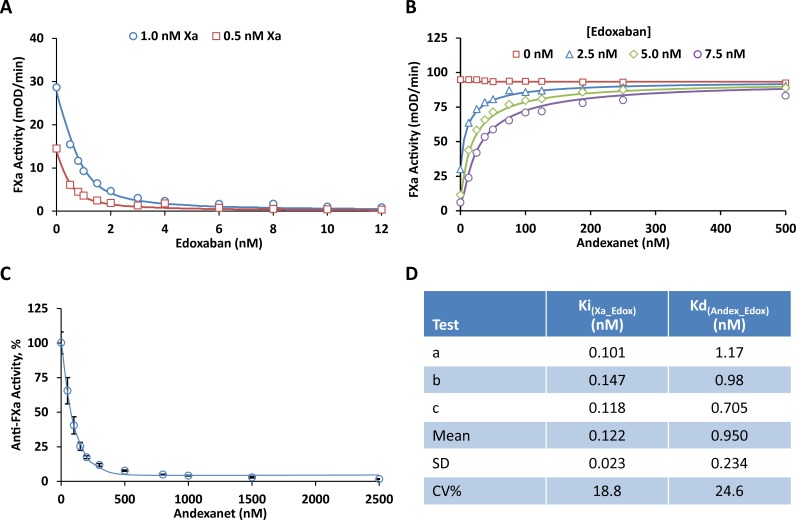
Edoxaban anti-FXa activity and andexanet effects *in vitro*. (A) Inhibition of FXa chromogenic activity by edoxaban in a buffer system with purified enzyme. Human FXa at 0.5 nM (□) and 1.0 nM (○) were pre-incubated with increasing concentrations of edoxaban (0, 0.5, 0.8, 1, 1.5, 2, 3, 4, 6, 8, 10, 12 nM) at RT for 2 hrs. Residual FXa activity was determined by measuring the initial rates of peptidyl substrate hydrolysis (mOD_405_/min) at RT over 5 min. The initial velocity was fitted with Dynafit to obtain the kinetic parameters Ki and kcat using the pre-determined Km (82.2 μM) as described in Materials and Methods. Fig 2A shows a representative result from one of the three experiments shown in Fig 2D (Test a for Ki). The symbols represent the measured mean initial rate from quadruplicate wells at each edoxaban concentration. The solid lines were drawn using the best fitted values with Ki = 0.101 nM, kcat = 156 1/s. (B) Reversal of edoxaban-induced inhibition of FXa chromogenic activity by andexanet in a buffer system with purified enzyme. Human FXa (3.0 nM); different concentrations of edoxaban at 0 (□), 2.5 (△), 5.0 (◇),and 7.5 nM (○); and increasing concentrations of andexanet (0, 12.5, 25, 37.5, 50, 75, 100, 125, 188, 250, 500 nM) were pre-incubated at RT for 2 hrs. Residual FXa activity was determined by measuring the initial rates of peptidyl substrate hydrolysis (mOD_405_/min) at RT over 5 min. The initial velocity was fitted with Dynafit to obtain the kinetic parameters Kd and kcat using the pre-determined Km (82.2 μM) and Ki (0.122 nM) as described in Materials and Methods. Fig 2B shows a representative result from one of the three experiments shown in Fig 2D (Test b for Kd). The symbols represent the measured mean initial rate from duplicate wells at each andexanet concentration. The solid lines were drawn using the best fitted values with Kd = 0.98 nM, kcat = 176 1/s. (C) Reversal of edoxaban-induced anti-FXa activity by andexanet in human plasma. Edoxaban (76 ng/mL, 0.136 μM) and increasing concentrations of andexanet (0, 0.05, 0.1, 0.15, 0.2, 0.3, 0.5, 0.8, 1, 1.5, 2.5 μM) were prepared in human plasma and pre-incubated at RT for 30 min. Residual anti-FXa activity for edoxaban was measured as described in Materials and Methods. Fig 2C shows edoxaban anti-FXa activity (%) after normalization of the results to the mean anti-FXa value at 0 nM andexanet. Data were plotted as the mean ± standard deviation from three separate experiments. (D) Constants for edoxaban interaction with FXa (Ki) and andexanet (Kd) determined by the kinetic measurements as described in panel (A) and panel (B), respectively.

The affinity of edoxaban for FXa was determined by measuring residual FXa activity in a buffered system (Fig 2A). The binding affinity between andexanet and edoxaban was then determined in the same assay (Fig 2B) using a range of edoxaban and andexanet concentrations. Finally, reduction of edoxaban-mediated anti-FXa activity was measured in human plasma upon addition of increasing concentrations of andexanet (Fig 2C). These assays showed that edoxaban has a high affinity for FXa, with a mean Ki in the sub-nanomolar range (Fig 2D). Andexanet demonstrated a high affinity for edoxaban (mean Kd, 0.95 nM) and dose-dependently reversed the edoxaban-mediated anti-FXa activity across the range of edoxaban concentrations (Fig 2D).

**Fig 3:**
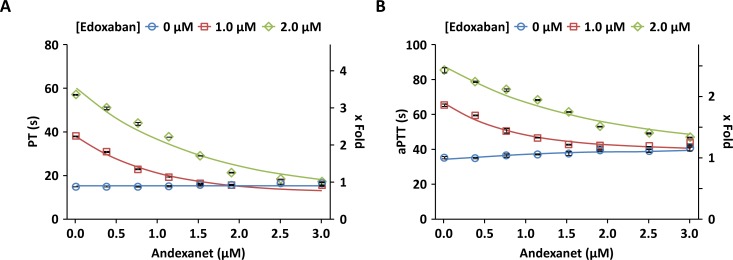
In vitro reversal of edoxaban-induced prolongation of PT and aPTT in human plasma. Plasma samples contained edoxaban at 0 (○), 1.0 (□), and 2.0 μM (◇) and increasing concentrations of andexanet (0, 0.38, 0.76, 1.14, 1.52, 1.9, 2.5, and 3.0 μM). PT and aPTT were measured on Stago Compact as described in Materials and Methods. Data were plotted as the mean ± standard deviation from two separate experiments.

**Fig 4:**
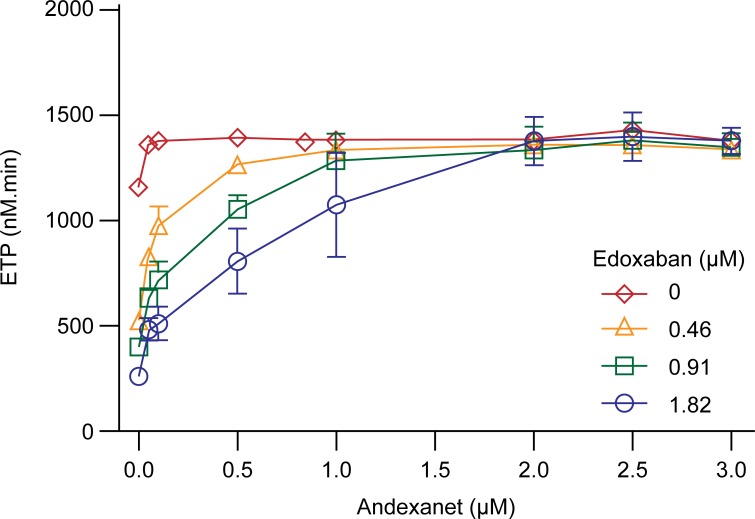
*In vitro* reversal of edoxaban-induced inhibition of thrombin generation in human plasma. Plasma samples contained edoxaban at 0 (◇), 250 (△), 500 (□), and 1000 (○) ng/mL (0, 0.46, 0.91, 1.82 μM) and increasing concentrations of andexanet (0, 0.05, 0.1, 0.5, 1.0, 2.0, 2.5, 3.0 μM). Data (ETP) were plotted as mean ± standard deviation from two separate experiments. Additional thrombin generation parameters are provided in S2 Fig.

Andexanet also dose-dependently reversed the prolongation of PT and aPTT mediated by edoxaban in human plasma (Fig 3), whereas minimal effect on PT (7.4%) or aPTT (14%) prolongation was observed with control plasma at the highest andexanet concentration (3 μM), possibly due to weak interaction of andexanet at high concentration with FVa in solution phase [26]. Additionally, edoxaban-induced inhibition of thrombin generation was dose-dependently reversed by andexanet at concentrations in molar excess relative to the edoxaban concentration (Fig 4). Edoxaban-induced changes in peak, time to peak, and lagtime (S1 Fig) were also reversed by andexanet (S2 Fig). These studies demonstrate that andexanet can reverse the anticoagulant effects of edoxaban *in vitro*, similar to what has been previously demonstrated with rivaroxaban, apixaban, and betrixaban [22].

### Rabbit model

#### Blood loss

**Fig 5:**
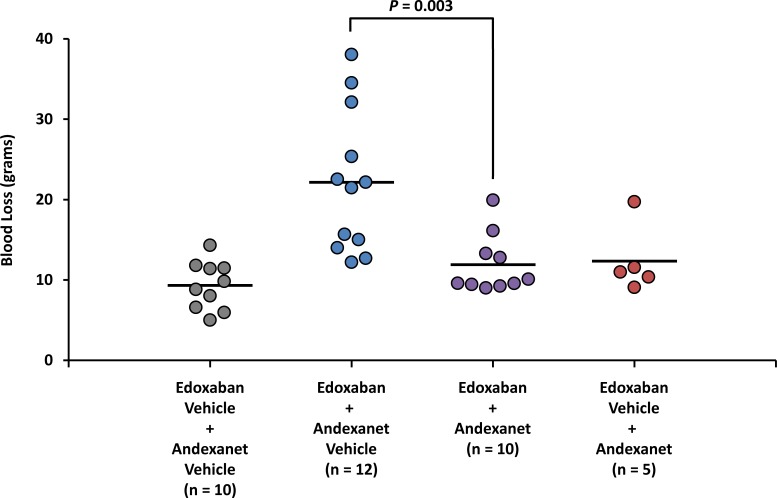
Total blood loss at end of study. The procedure for the rabbit liver injury prophylactic blood loss model was a modification of a previously published hepatosplenic liver injury model as described in Materials and Methods. Rabbits were administered edoxaban (1 mg/mL) or edoxaban vehicle at Time 0. Andexanet or andexanet vehicle was administered IV over 5 minutes (from Time 20 to Time 25 minutes). A standardized liver injury was made into 2 liver lobes with 5 incisions in each lobe and allowed to bleed for 15 minutes (from Time 25 to Time 40 minutes). The blood loss was measured in grams by weighing pre-weighed dry gauze. Each data point represents the measurement from an individual rabbit at Time 40 minutes (15 minutes after liver injury). Horizontal bars represent mean values.

Mean blood loss increased ~2.4-fold in rabbits anticoagulated with edoxaban (edoxaban + andexanet vehicle) vs. non-anticoagulated rabbits (edoxaban vehicle + andexanet vehicle; 22.2 ± 8.9 vs. 9.3 ± 3.0 g; *P* = 0.0003; Fig 5); the increase in median blood loss was ~2.3-fold. Following andexanet administration in edoxaban-anticoagulated rabbits (edoxaban + andexanet), mean blood loss decreased to 11.9 ± 3.7 g, a level comparable to that observed in non-anticoagulated rabbits (9.3 ± 3.0 g; *P* > 0.05; Fig 5); median blood loss decreased to 9.8 g. After adjusting for blood loss in non-anticoagulated rabbits, this decrease following andexanet administration translated to an 80% (~1.9-fold) reduction in mean blood loss compared with andexanet vehicle (2.6 vs. 12.9 g, respectively; *P* = 0.003); the median reduction in blood loss was 96%.

#### Pharmacodynamic parameters

**Fig 6:**
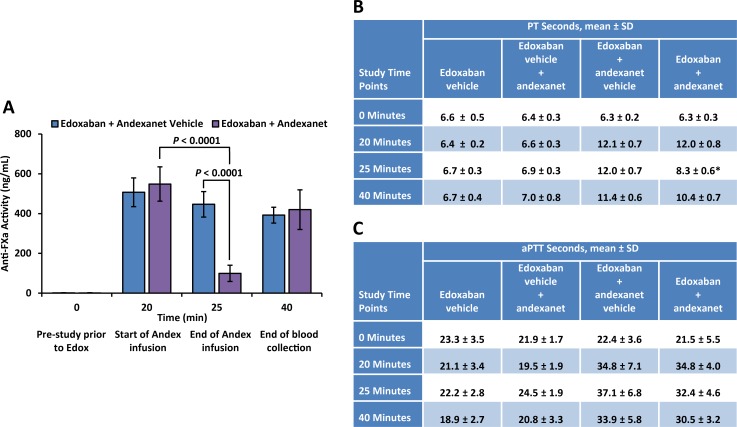
Change in pharmacodynamic parameters following administration of andexanet in edoxaban-anticoagulated rabbits. (A) Edoxaban anti-FXa activity was measured by a modified anti-FXa activity assay. The anti-FXa activity in unknown samples was quantified using the known concentrations of edoxaban in rabbit plasma as standards and expressed as ng/mL. Data are plotted as mean ± standard deviation. (B) PT measurements in seconds. (C) aPTT measurements in seconds. Determination of PT and aPTT was performed on an Instrumentation Laboratory ACL Elite. HemosIL PT-Fibrinogen kit was used for PT measurements, and HemosIL SynthASil kit was used for aPTT measurements.

Andexanet rapidly reduced anti-FXa activity associated with edoxaban anticoagulation by 82% (from 548 ± 87 to 100 ± 41 ng/mL; a decrease of 449 ng/mL) at the end of the 5-minute andexanet infusion (Time 25 minutes) compared with that observed at the beginning of the andexanet infusion (Time 20 minutes; *P* < 0.0001 for the difference between Time 20 and 25 minutes; Fig 6A). In contrast, in edoxaban-anticoagulated rabbits administered andexanet vehicle, anti-FXa activity was reduced by 12% (from 507 ± 72 to 447 ± 64 ng/mL; a decrease of 60 ng/mL; *P* = 0.04 for the difference between Time 20 and 25 minutes), and edoxaban levels continued to decline slowly over the remaining 15 minutes of the experimental period.

Andexanet also significantly corrected the prolongation of PT in rabbits anticoagulated with edoxaban (Fig 6B). Edoxaban increased mean PT from baseline by 1.9-fold, and andexanet shortened the mean PT by 31% (*P* < 0.001) at the end of andexanet infusion (Time 25 minutes) relative to that observed at the beginning of andexanet infusion (Time 20 minutes). The prolongation of aPTT in rabbits anticoagulated with edoxaban was not corrected by andexanet (Fig 6C). Edoxaban increased mean aPTT from baseline by 1.6-fold, and andexanet decreased the mean aPTT by ~7% (not statistically significant) at the end of andexanet infusion (Time 25 minutes) relative to that observed at the beginning of andexanet infusion (Time 20 minutes).

#### Edoxaban and andexanet plasma levels

**Fig 7:**
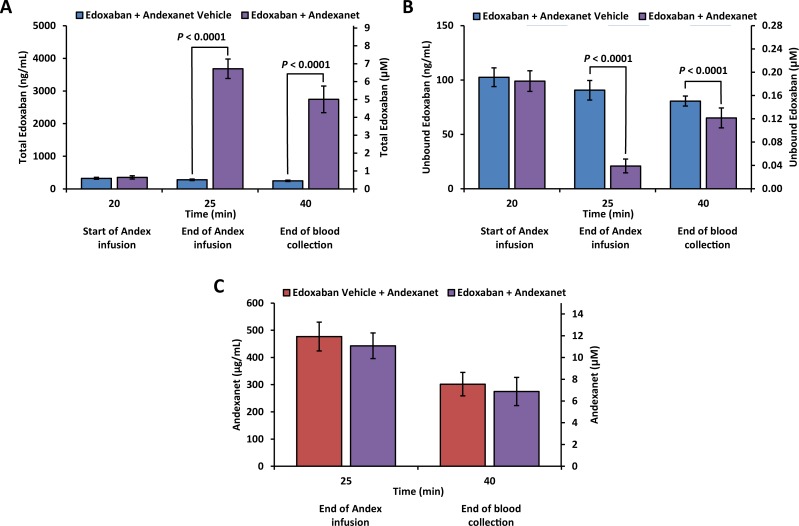
Change in pharmacokinetic parameters following administration of andexanet in edoxaban-anticoagulated rabbits. (A) Total edoxaban plasma concentration. (B) Unbound edoxaban plasma concentration. Total and unbound (pharmacologically active) edoxaban plasma levels were determined by LC/MS/MS with a turbo-ion spray source using an internal standard (edoxaban-D6) as described in Materials and Methods. Pharmacologically active unbound edoxaban was separated using an HTD96b apparatus at 37°C for approximately 4 hours with gentle vortexing. (C) Andexanet plasma concentration was determined by ELISA with paired antibodies recognizing human FX/FXa. Andexanet standard was freshly prepared in blocking buffer with the same lot material used in this study for 8-point curve (0–200 ng/mL). Data are plotted as mean ± standard deviation.

Mean total edoxaban plasma concentration increased significantly following andexanet administration compared with andexanet vehicle, and remained elevated at study end (*P* < 0.0001 at Time 25 and 40 minutes), reflecting redistribution of edoxaban from the extravascular space into plasma due to high affinity binding of edoxaban to andexanet (Fig 7A). Mean unbound edoxaban plasma concentration decreased by ~80% (from 99 ± 10 to 21 ± 6 ng/mL) immediately following the end of andexanet administration (Time 25 minutes) compared to that prior to andexanet administration (Time 20 minutes). In contrast, administration of andexanet vehicle did not change total or unbound edoxaban plasma concentration. The difference in mean unbound edoxaban plasma concentration between edoxaban + andexanet vs. edoxaban + andexanet vehicle groups at Time 25 minutes was statistically significant (21 vs. 91 ng/mL; *P* < 0.0001). The unbound edoxaban concentration began to increase at study end (mean unbound edoxaban at Time 40 minutes of 65 ng/mL), reflecting the clearance of andexanet (Fig 7B).

Mean andexanet plasma concentrations in the edoxaban and edoxaban vehicle-treated rabbits were similar (10.8 and 11.6 μM, corresponding to 443 and 477 μg/mL, respectively) immediately following administration of andexanet (Time 25 minutes, end of infusion) (Fig 7C). At study end (Time 40 minutes; 15 minutes after completing andexanet administration), the mean andexanet plasma concentration had decreased to ~7 μM (275 and 302 μg/mL) in both the edoxaban and edoxaban vehicle-treated rabbits, respectively, as the drug was cleared. Based on molar concentration of andexanet (10.8 μM) and edoxaban (6.7 μM), the molar ratio of andexanet to total edoxaban was 1.6:1 at the end of andexanet administration (Time 25 minutes), demonstrating a molar excess of andexanet.

#### Correlations between blood loss and anti-FXa activity

**Fig 8:**
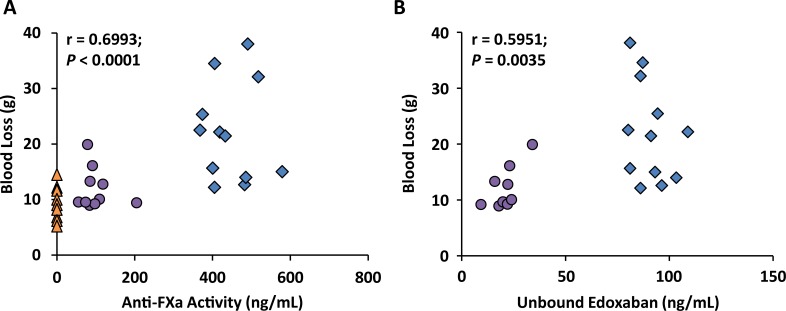
Correlation between blood loss and pharmacodynamic markers in edoxaban-anticoagulated rabbits. Blood loss correlated with (A) anti-FXa activity and (B) unbound edoxaban plasma concentrations. Each data point represents the measurements from an individual rabbit at the end of andexanet infusion (Time 25 minutes) for anti-FXa and unbound edoxaban, and 15 minutes after liver injury for blood loss (Time 40 minutes). △ vehicle alone (both edoxaban + andexanet vehicle); ○ edoxaban + andexanet; ◇ edoxaban + andexanet vehicle.

The observed 80% reduction in mean blood loss following andexanet administration correlated with a 449-ng/mL (82%) decrease in anti-FXa activity (r = 0.6993, *P* < 0.0001) (Fig 8A) and an ~80% decrease in unbound edoxaban plasma concentration (r = 0.5951, *P* = 0.0035) (Fig 8B) immediately following the end of andexanet administration at Time 25 minutes.

## Discussion

Edoxaban is the newest FXa inhibitor approved for treatment of VTE and stroke prevention, and the availability of clinical data with andexanet is limited. Therefore, we investigated edoxaban in a rabbit model of acute hemorrhage to characterize both *in vitro* interaction between andexanet and edoxaban, and *in vivo* effects of andexanet on blood loss in rabbits anticoagulated with edoxaban. The results of this study allowed better understanding of differences between edoxaban and other FXa inhibitors and the potential class effect of andexanet on reversing the anticoagulation of all FXa inhibitors.

The *in vitro* assays, using either a buffer system with purified enzyme or human plasma, demonstrated that andexanet bound to edoxaban with high affinity. The andexanet binding affinity for edoxaban (Kd, 0.95 nM) was similar to that observed for other direct FXa inhibitors (rivaroxaban Kd, 1.53 nM; apixaban Kd, 0.58 nM, betrixaban Kd 0.53 nM) [22]. This high-affinity binding allows andexanet to reverse edoxaban-induced anticoagulant activity in a dose-dependent manner at clinically relevant concentrations [27]. Consequently, andexanet reversed the edoxaban-mediated anti-FXa activity and other coagulation pharmacodynamic parameters *in vitro*. In the *in vitro* studies, andexanet also restored normal thrombin generation (reflected as the increase in endogenous thrombin potential or ETP) in human plasma containing high levels of edoxaban (up to 1.82 μM) to the levels observed without edoxaban. In the absence of edoxaban, thrombin generation (ETP) increased by approximately 20% after addition of andexanet to human plasma, an anticipated effect due to the known interaction of andexanet with tissue factor pathway inhibitor (TFPI) in the plasma; this effect is accentuated when thrombin generation is initiated by low concentrations of tissue factor [28]. Notably, the increase in ETP reached a plateau at 50 nM andexanet, and no additional increase in ETP was observed at andexanet concentrations up to 3 μM.

The andexanet-TFPI interaction may enhance TF-initiated thrombin generation and contribute to transient elevations in coagulation markers (e.g., D-Dimer and prothrombin fragments 1 and 2) [28, 29]. However, no evidence of thrombotic events after receiving andexanet was observed in preclinical monkey toxicology studies [30] or in Phase 2 and 3 clinical trials in healthy subjects [31, 32]. Similarly, clinical trials evaluating the TFPI-binding antibody, concizumab, also showed elevations in coagulation markers, without evidence of thrombotic events [33]. In the initial interim safety analysis of 67 bleeding patients from the ongoing phase 3b/4 trial (ANNEXA-4), 18% of patients experienced thrombotic events [23]. Importantly, only 1 patient had resumed therapeutic anticoagulation prior to the thrombotic event. In an updated safety analysis in 105 bleeding patients, more patients (40%) resumed anticoagulation after the bleeding event, and the rate of thrombotic events was 12% [24].

In a rabbit model of acute hemorrhage, rapid reduction in anti-FXa activity and unbound (pharmacologically active) edoxaban concentration were associated with a decrease in blood loss in edoxaban-anticoagulated rabbits administered andexanet. The observation that total edoxaban plasma concentration *in vivo* increased after administration of andexanet to anticoagulated rabbits reflects the redistribution of edoxaban from the extravascular space into plasma. This finding is expected and consistent with andexanet’s mechanism of action. Similar results were observed for total rivaroxaban plasma concentrations in the previous rabbit study [22], and in Phase 3 clinical studies in older healthy subjects [31].

In the previous study using the same rabbit model of acute hemorrhage [22], andexanet showed a slightly greater ability to reverse the anticoagulation activity of rivaroxaban; it decreased anti-FXa activity and unbound rivaroxaban plasma concentration by 98% (compared with ~80% in this study). However, andexanet showed a similar ability to reduce blood loss in both studies; it reduced blood loss by more than 85% in rivaroxaban-anticoagulated rabbits and by 82% in edoxaban-anticoagulated rabbits. These data suggest that >90% reversal of coagulation biomarkers by andexanet may not be necessary to achieve a clinically meaningful reduction in blood loss.

In this study, a molar ratio of 1.6:1 (andexanet to edoxaban) significantly decreased anti-FXa activity and blood loss associated with edoxaban treatment. This is consistent with previous observation when ≥1.3:1 molar ratio was sufficient to reverse the anticoagulant effects of rivaroxaban in the same animal model [22]. The correlation of anti-FXa activity with blood loss provides additional support for use of anti-FXa activity as a clinical biomarker likely to predict the clinical benefit of andexanet for reversal of bleeding due to FXa inhibitor-mediated anticoagulation. In a Phase 2 clinical study in edoxaban-anticoagulated healthy volunteers, an 800-mg bolus of andexanet reduced anti-FXa activity by 73%, which was sustained during a subsequent 1-hour infusion of andexanet (8 mg/min) and returned to pretreatment levels approximately 2 hours after the end of infusion [34]. In addition, the unbound fraction of edoxaban was decreased, and thrombin generation was restored to within the baseline range for 2 hours. Similar effects of andexanet on pharmacodynamic markers were demonstrated in two Phase 3 studies with apixaban and rivaroxaban [31]. The sustained effects of andexanet on coagulation pharmacodynamic parameters during and post infusion are consistent with its half-life. The half-life of andexanet in rats, rabbits, and mice is 15 to 20 minutes; the half-life in monkeys and humans is 40 to 45 minutes. The correlation between blood loss and anti-FXa activity in preclinical *in vivo* blood loss models further supports the use of anti-FXa activity as a biomarker for assessing anticoagulation reversal in clinical trials.

The strengths of this study include blinding of the surgical technician to the treatment group and the established relevance of this model to bleeding associated with surgery in humans [35]. Limitations of this study include the short duration of bleeding assessment (15 minutes) in the rabbit blood loss model.

Non-specific replacement of clotting factors with PCCs have been investigated in rabbit models of acute bleeding using edoxaban, as well as rivaroxaban and apixaban. Reduction in blood loss or improvement in coagulation parameters was partial and inconsistent [16–18]. Similarly, in healthy volunteers anticoagulated with edoxaban or rivaroxaban, PCCs corrected some, but not all, clinical coagulation parameters, and data on bleeding from punch biopsy showed only partial reduction in blood loss [8, 14, 15]. Given the limited data and lack of consistent effects on bleeding and coagulation parameters, the clinical value of PCCs for management of bleeding events in patients anticoagulated with FXa inhibitors is unclear, especially since there are no data from controlled clinical trials to support their use in this indication. Several guidelines for the management of life-threatening bleeding recommend that, until specific reversal agents are available, clinicians should consider the use of PCCs in bleeding patients anticoagulated with FXa inhibitor when immediate hemostatic support is required [36–42].

In summary, andexanet rapidly and significantly reduced the unbound, pharmacologically active fraction of edoxaban, rapidly reduced anti-FXa activity and normalized (or partially normalized) other coagulation PD biomarkers *in vitro*. *In vivo*, in edoxaban-anticoagulated rabbits, andexanet rapidly reduced anti-FXa activity that was correlated with reduction in blood loss. These data are in agreement with the mechanism of action of andexanet, which acts as a FXa decoy molecule that binds with high affinity and sequesters the FXa inhibitor in plasma, thereby allowing the return of endogenous FXa activity [22] and restoring normal hemostatic mechanisms as demonstrated in animal models of bleeding. Thus, the rabbit liver laceration model provides compelling evidence that andexanet can potentially achieve clinically meaningful reversal of anticoagulation mediated by FXa inhibitors and, consequently, reduce bleeding associated with FXa inhibitors. An ongoing andexanet clinical study in patients anticoagulated with FXa inhibitors who experience acute major bleeding will provide real-world efficacy and safety information in this patient population [NCT02329327] [23, 24, 43].

## Conclusions

Andexanet bound edoxaban with high affinity in ≤1.6:1 molar ratio that was sufficient to rapidly and dose-dependently reverse the effects of edoxaban on blood loss in a rabbit model of bleeding, as well as FXa activity and coagulation pharmacodynamic parameters *in vitro* and *in vivo*. These data support the ongoing clinical evaluation of andexanet for the management of acute major bleeding in patients receiving edoxaban, and future evaluations of andexanet efficacy and safety in the setting of surgery-related bleeding. The correlation between blood loss and anti-FXa activity supports the use of anti-FXa activity as a parameter for assessing anticoagulation reversal in clinical trials.

## Supporting information

S1 File Methods. (DOCX)

S1 Fig Inhibition of TF-initiated thrombin generation by edoxaban in human plasma. (DOCX)

S2 Fig Reversal of edoxaban-induced inhibition of thrombin generation by andexanet. (DOCX)
