# Supplementary material for: Andexanet alfa effectively reverses edoxaban anticoagulation effects and associated bleeding in a rabbit acute hemorrhage model
Source: PLoS One. 2018 Mar 28;13(3):e0195122. doi: 10.1371/journal.pone.0195122 (PMC5874076; doi:10.1371/journal.pone.0195122)
Supplement: S1 File — (DOCX) [file pone.0195122.s001.docx]

**S1 File. Methods**

The plasma concentration of edoxaban was quantified using a standard liquid chromatography, tandem mass spectrometry (LC/MS/MS) assay as described below. The percentage plasma bound (PPB) was determined using rapid equilibrium dialysis. The final unbound concentration was determined by multiplying the percent unbound by the total concentration.

**Total edoxaban concentration method**

Liquid-liquid extraction (LLE) and HPLC/MS/MS were used to determine the concentration of edoxaban in rabbit sodium citrate plasma. An aliquot of the extract was injected onto an HPLC/MS/MS triple quadrupole mass spectrometer. A Discovery HS-C18, 3-m high performance liquid chromatography (HPLC) column (2.1 × 50 mm, Supelco) was used to separate edoxaban and the internal standard, D6-edoxaban, from interfering compounds that may have been present in the sample extract. The peak area of the product ion of edoxaban was measured against the peak area of the product ion of the internal standard. A calibration curve ranging from 1.00 ng/mL to 1000 ng/mL was used to quantify edoxaban in the samples.

**Rapid equilibrium dialysis method**

A 96-well Micro-Equilibrium Dialysis Device from HTC (HTD96b) was utilized to determine the protein binding of edoxaban in rabbit plasma. Sodium citrate plasma spiked with edoxaban was placed in a chamber of the dialysis device and phosphate-buffered saline (PBS) was placed in the adjacent chamber. The plasma and buffer chambers were separated by a regenerated cellulose–molecular weight cutoff (MWCO) 12-14 KDa membrane. The device was then placed in an incubator set at 37°C and gently vortexed until equilibrium between the two chambers was reached. The plasma chamber and buffer chamber were then extracted and analyzed in order to determine the concentration of edoxaban in each chamber. The samples removed from the buffer chamber were diluted 1:1 with rabbit sodium citrate plasma prior to extraction. The samples removed from the plasma chamber were analyzed using the HPLC/MS/MS assay described above.
