# Supplementary material for: Andexanet alfa effectively reverses edoxaban anticoagulation effects and associated bleeding in a rabbit acute hemorrhage model
Source: PLoS One. 2018 Mar 28;13(3):e0195122. doi: 10.1371/journal.pone.0195122 (PMC5874076; doi:10.1371/journal.pone.0195122)
Supplement: S1 Fig — (DOCX) [file pone.0195122.s002.docx]

**S1 Fig. Inhibition of TF-initiated thrombin generation by edoxaban in human plasma**

a. Time-course profiles

b. Change of TF-CAT parameters as a function of edoxaban concentrations

TF-initiated thrombin generation was measured using the PPP-reagent (5 pM TF) as described under Material and Methods.

a. Time-course profiles of thrombin generation in pooled human plasma contained edoxaban, starting at 1000 ng/mL (1.82 µM) followed by 2-fold seral dilutions. The figure shows the representative results from one of the two experiments.

b. Change of TF-CAT parameters as a function of edoxaban concentrations for ETP, Peak, Lag Time, Time-to-Peak (TTP), and Velocity index (VelIndex), respectively. Data are shown as Mean±SD from two experiments.
