# Supplementary material for: Andexanet alfa effectively reverses edoxaban anticoagulation effects and associated bleeding in a rabbit acute hemorrhage model
Source: PLoS One. 2018 Mar 28;13(3):e0195122. doi: 10.1371/journal.pone.0195122 (PMC5874076; doi:10.1371/journal.pone.0195122)
Supplement: S2 Fig — (DOCX) [file pone.0195122.s003.docx]

**S2 Fig.** **Reversal of edoxaban-induced inhibition of thrombin generation by andexanet**

a. Time-course profiles

b. Change of TF-CAT parameters as a function of edoxaban and andexanet concentrations

Human plasma contained edoxaban (0, 0.25, 0.5, 1.0 µg/mL; corresponding to 0, 0.46, 0.91, 1.82 µM) and increasing concentrations of andexanet (0, 0.05, 0.1, 0.5, 1.0, 2.0, 2.5, 3.0 µM).

a. Time-course profiles of thrombin generation. The figure shows the representative results from one of the two experiments.

b. Reversal of edoxaban-induced inhibition of thrombin generation by andexanet. Change of thrombin generation parameter versus andexanet concentration is shown in each panel with Peak, Lag Time, Time-to-Peak (TTP) and Velocity index (VelIndex), respectively. Data are shown as Mean±SD from two experiments.
